# Regional Variations in Health Behavior Structures: A Social Determinants of Health Approach

**DOI:** 10.3390/healthcare13202557

**Published:** 2025-10-10

**Authors:** Seungman Lee, Sungho Yoon, Hanbeom Kim

**Affiliations:** 1 Department of Sport Science, Hankyong National University, Anseong 17579, Republic of Korea; lsm14pe@hknu.ac.kr; 2 Department of Physical Education, Kangwon National University, Chuncheon 24341, Republic of Korea; peysh7567@kangwon.ac.kr

**Keywords:** social determinants of health theory, health behavior action model, regional differences analysis, multi-group structural equation modeling

## Abstract

**Background/Objectives:** This study analyzes how Health and Fitness Awareness influences quality of life (QOL), mediated by Health Behavior Action and Safety Behavior Practice, within the Social Determinants of Health (SDH) framework. **Methods:** Accordingly, a multi-group structural equation modeling (SEM) analysis was conducted on the data obtained from 6601 respondents selected from the 2024 National Sports for All Survey, jointly administered by the Ministry of Culture, Sports and Tourism and Korea Sports Promotion Foundation. Nationally representative survey data was collected across metropolitan, mid-sized, and rural areas in South Korea. The analysis further examined whether the structural pathways differed by regional size. Outcome measures included path coefficients and latent mean differences among Health and Fitness Awareness, Health Behavior Action, Safety Behavior Practice, and Improvement in QOL. **Results:** The analysis revealed that Health and Fitness Awareness significantly influenced both Health Behavior Action and Safety Behavior Practice; these, in turn, had positive effects on Improvement in QOL. Moreover, the structural pathways differed by region: whereas Health Behavior Action played a more significant mediating role in large cities, Safety Behavior Practice was more prominent in mid-sized ones. **Conclusions:** These findings are expected to provide a theoretical and policy-based foundation for region-specific health promotion strategy development and health equity advancement.

## 1. Introduction

Advancements in medicine and public health services have ensured human prosperity over the years. However, the human desire for extended longevity remains a critical concern. In contemporary society, health is no longer defined merely as the absence of disease or infirmity. It is now considered a key determinant of quality of life (QOL), encompassing social participation in daily life, emotional stability, and psychological recovery [1,2]. Accordingly, the paradigm of health has shifted from “how long one lives” to “how well one lives in good health.”

In its landmark report, the Commission on Social Determinants of Health of the World Health Organization (WHO) asserts that “health inequities will remain unless the conditions in which people live are improved” and frames the issue as a matter of social justice [3]. Health status disparities are structurally recurring phenomena that occur both between and within countries—between urban and rural areas, core and peripheral regions, and high- and low-income populations [4,5]. South Korea, where the population concentration in the capital region remains a major social issue, is no exception. Substantial differences in health behaviors and access to healthcare infrastructure exist between metropolitan and rural areas. In particular, the country exhibits significant regional disparities in physical activity participation rates, health information access, and community-based health support system availability. These disparities can have long-term effects on individuals’ Health Behavior Action and Improvement in QOL [6,7,8].

Individual health behavior is shaped not only by personal traits but also by structural conditions. The WHO highlights that physical activity is strongly associated with social factors such as income, education, gender, and race, and calls for policy interventions through the Social Determinants of Health (SDH) framework [3].

The SDH framework emphasizes that health disparities are shaped by social, economic, and political conditions, many of which can be modified through policy interventions. It highlights that these contextual factors—such as income, education, employment, housing, and access to health resources—create the “causes of the causes” of inequality by influencing individuals’ opportunities to engage in healthy behaviors, including physical activity [1,3,5,9].

Based on this perspective, the current study seeks to examine disparities in Health Behavior Action by considering not only individual-level characteristics but also structural, social, and regional factors influencing exercise participation.

How severe are regional health disparities in South Korea? Specifically, to what extent do differences in health status and Health Behavior Action exist among large-sized cities, mid-sized cities, and rural areas? Recent studies suggest that health behaviors vary significantly across regions in South Korea, intensifying the health inequality issue [10,11]. In large cities, particularly in the capital region, indicators such as participation in health screenings, walking practice rates, nutritional awareness, and adherence to safety guidelines are relatively favorable. In contrast, mid-sized cities and rural areas experience structural disadvantages in accessing health information, participating in preventive health programs, and utilizing emergency and mental health services [12].

Chae and Han [13] found that disparities in health behavior between urban and rural youth in Korea emerged as early as adolescence; this finding highlights the structural dimension of health inequality. Similarly, Lee [14] indicated that regional disparities in access to medical services significantly influence individuals’ engagement in Health Behavior Action. Furthermore, Kim and Ruger [15] reported that health behavior-related inequalities based on socioeconomic status and gender are particularly high in South Korea.

Health disparities arise from unequal access to health resources, welfare infrastructure, and information, as well as from differences in social status, gender, and cultural perceptions. These inequalities affect both individual attitudes toward health and the structural processes shaping health behaviors. Recognizing these challenges, international organizations and national health policies increasingly emphasize the SDH framework, underscoring the need for empirical studies that analyze region-specific pathways of health inequality [3,12].

The SDH framework enables a structural interpretation of health that extends beyond individual responsibility. It attributes regional health disparities to environmental constraints and clarifies how contextual and structural conditions fundamentally influence health outcomes [1]. However, most studies to date [10,11,13,14,15,16] have taken a micro-level approach, focusing mainly on individual attitudes, knowledge, or behavioral changes. In contrast, research that integrates external factors such as social structures and regional environments remains limited. Furthermore, studies addressing regional health disparities tend to rely on descriptive statistics, such as QOL indicators or differences in healthy life expectancy, or treat regional characteristics as mere control variables [4,5]. Consequently, quantitative analyses revealing structural causal relationships grounded in the SDH framework remain insufficient.

Additionally, many studies consider Health Behavior Action a single-dimensional variable or fail to conceptualize health and fitness awareness as a mediating factor. Consequently, the mechanisms linking health behaviors and improvement in QOL are not adequately clarified. Nevertheless, a recent study by Chang, Park, and Lee [17] provided empirical evidence that satisfaction with local communities’ physical environment significantly influences individuals’ physical activity and perceived health. These findings highlight the need to examine the relationship between health behaviors and QOL within a structural framework incorporating regional environmental factors.

This study applies the SDH framework to model the relationships among health and fitness awareness, Health Behavior Action, Safety Behavior Practice, and QOL, comparing these pathways across regional types to empirically identify the internal variations in health behavior structures by region: large-sized cities, mid-sized cities, and rural areas. Structural equation modeling (SEM) was used to investigate each pathway’s significance and explanatory power, differences in path coefficients, and latent mean differences to empirically identify the internal variations in health behavior structures by region. This approach allows for an understanding of how regional contexts shape individual health outcomes and provides a novel contribution by integrating SEM with region-specific analyses.

This study analyzes how individual health behavior is shaped by the intersection of social–structural constraints and psychological judgments, providing a theory- and policy-based foundation to address health inequalities. It conceptualizes health as the product of social, personal, and cultural factors, and offers an empirical basis for mitigating regional health disparities through an analytical framework and practical strategies.

### 1.1. Research Purpose and Conceptual Model

**Figure 1:**
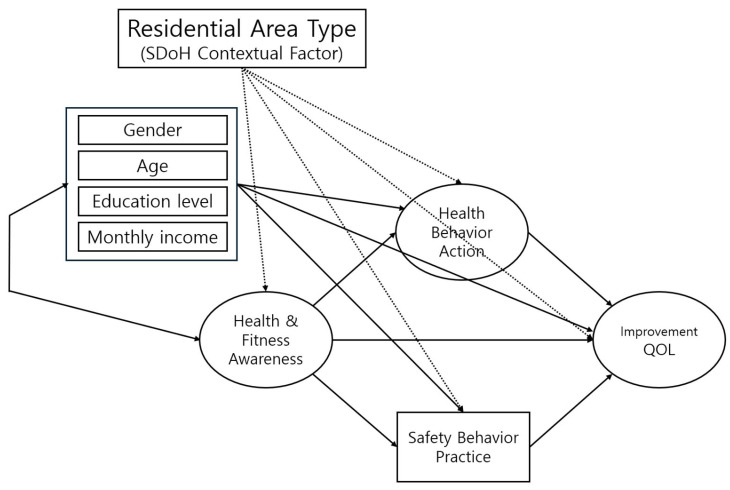
Hypothetical Model.

This study’s purpose was to empirically examine the structural relationships among factors influencing individual Health Behavior Action based on the theoretical SDH framework. Specifically, this study analyzes how social and perceptual factors affect health behaviors and examines whether these structural pathways differ across regions. To this end, SEM was conducted to investigate the relationships among Health & Fitness Awareness, Health Behavior Action, and Safety Behavior Practice. In alignment with the Social Determinants of Health (SDoH) framework, this study hypothesizes that Residential Area Type (large cities, mid-sized cities, and rural areas) acts as a structural moderator that shapes the hypothesized relationships between constructs. Specifically, Multigroup Structural Equation Modeling (SEM) was employed to test how the regional macro-context differentially impacts the strength and significance of the pathways connecting awareness, behaviors, and QOL improvement. This approach allows us to identify the structural basis of health inequality and propose regionally tailored policy responses. Figure 1 depicts the conceptual model used in this study.

### 1.2. Research Hypotheses

This study examines the following hypotheses:

**H1-1.**  Health and Fitness Awareness has a direct effect on Improvement in QOL.

**H1-2.**  Health and Fitness Awareness has a direct effect on Health Behavior Action.

**H1-3.**  Health Behavior Action has a direct effect on Improvement in QOL.

**H1-4.**  Health Behavior Action mediates the relationship between Health and Fitness Awareness and Improvement in QOL.

**H2-1.**  Health and Fitness Awareness has a direct effect on Safety Behavior Practice.

**H2-2.**  Safety Behavior Practice has a direct effect on Improvement in QOL.

**H2-3.**  Safety Behavior Practice mediates the relationship between Health and Fitness Awareness and Improvement in QOL.

**H3.**  The structural pathways of the Health Behavior Action Model differ significantly across regions (large cities, mid-sized cities, and rural areas).

## 2. Materials and Methods

### 2.1. Participant Details

This study used raw data collected as part of the 2024 National Sports for All Survey. The survey was supervised by the Ministry of Culture, Sports, and Tourism and the Korea Sports Promotion Foundation. The target population was all Korean residents aged 10 years and older, excluding individuals living in dormitories, special institutions (e.g., military, prisons, long-term care facilities), or abroad at the time of the survey. To ensure national representativeness, the sampling design applied stratification by region (17 metropolitan/provincial areas), sex (male/female), and age group (10 s, 20 s, 30 s, 40 s, 50 s, 60 s, and 70+). However, equal allocation by urbanicity (large cities, mid-sized cities, rural areas) was not applied in the original survey design. Weights were not applied in the present analysis; instead, the full raw dataset was used. In this study, large cities were defined as Seoul Metropolitan City and metropolitan cities with populations over one million; mid-sized cities referred to other urban areas designated as “dong”; and rural areas referred to towns and villages (“eup/myeon/ri”) that had not been upgraded to “dong” under urban development policies. Although we use the term ‘region’ in the administrative sense (large city, mid-sized city, rural area), the core dimension we are exploring is the variation in health behavior structures across the continuum of development concentration (urban–rural differences), rather than distinct geographical or political segments. The survey included items on physical activity practices, health-related lifestyle behaviors, sports facility utilization, and perceptions related to health and sports. For the present study, we analyzed the full raw dataset of the 2024 National Sports for All Survey. After excluding respondents with missing data and those not reporting regular participation in physical activities, the final analytic sample comprised 6601 individuals (Table 1).

### 2.2. Measures

This study used selected items from the 2024 National Sports for All Survey that aligned with its research objectives. The survey instrument was developed by the Ministry of Culture, Sports, and Tourism and the Korea Sports Promotion Foundation based on previous national surveys and official guidelines. The questionnaire was reviewed by experts and pre-tested prior to nationwide administration. Data collection was conducted by professional survey agencies using a stratified sampling design across all 17 metropolitan and provincial regions, which ensured coverage of both urban and rural populations. The relevant measurement instruments comprise five major domains. First, demographic characteristics included gender, age group, educational attainment, monthly income, and residential areas. Second, the Health and Fitness Awareness domain comprised items that assessed individuals’ perceived health status and physical fitness levels. For example, respondents were asked, “How would you rate your current level of health?” and “How would you rate your current level of physical fitness?” Third, Health Behavior Action was assessed using items related to the degree of health and fitness maintenance behaviors, including regular participation in physical activity, reception of adequate rest and sleep, access to a balanced diet and nutritional supplementation, and avoidance of smoking and alcohol consumption. Example items included: “During a typical week, how often do you engage in regular physical activity?” and “Do you usually obtain sufficient sleep and rest?” Fourth, Safety Behavior Practice was measured using items assessing respondents’ engagement in safety-related behaviors during physical activity. For instance, “Do you wear safety equipment (e.g., helmet, protective gear) when participating in physical activities?” Fifth, Improvement in QOL was measured using items capturing the positive effects of regular physical activity perceived by individuals, including the maintenance of physical health, enhancement of mental health, support for daily functioning, and reduction in medical expenses. For example, “Since engaging in regular physical activity, have you experienced improvements in your physical health?” All items were rated on a 5-point Likert scale (1 = not at all true, 5 = very true), which enabled the quantification of responses as continuous variables.

### 2.3. Data Analysis Procedures

The collected data were analyzed using SPSS 18.0 (SPSS Inc., Chicago, IL, USA) and Mplus 8.0 (IBM Corp., Armonk, NY, USA). All statistical analyses adhered to accepted SEM standards for model specification and validation. Prior to conducting the main analysis, missing values and outliers were identified and removed using a data-cleaning process. Normality assumptions were assessed using skewness and kurtosis, and all values were within acceptable ranges for SEM analysis. Subsequently, respondents were categorized into three regional groups based on administrative district classification variables: large-sized cities, mid-sized cities, and rural areas. Descriptive statistics and correlation analyses were conducted to examine the distribution and interrelationships between the variables. Furthermore, confirmatory factor analysis (CFA) was performed to assess the construct validity of the latent variables, and Cronbach’s α coefficients were used to assess their reliability. Subsequently, SEM was used to analyze the pathways through which Health Behavior Action and Safety Behavior Practice, mediated by Health and Fitness Awareness, influence the Improvement in QOL. The significance of the mediation effects was tested using the bootstrapping method. Model fit was evaluated using the Comparative Fit Index (CFI), the Tucker–Lewis Index (TLI), the Root Mean Square Error of Approximation (RMSEA), and the Standardized Root Mean Square Residual (SRMR). Following conventional guidelines [18,19], values of CFI and TLI > 0.90, RMSEA < 0.08, and SRMR < 0.08 were considered acceptable, while CFI and TLI > 0.95 and RMSEA < 0.05 indicated excellent fit. Finally, a multigroup SEM analysis was conducted to examine structural differences across regions. This analysis tested the invariance of the path coefficients and compared the structural differences in the Health Behavior Action model across the three regional groups.

## 3. Results

### 3.1. Correlation Analysis Among Measured Variables by Region

As shown in Table 2, the correlation analysis of the study’s key variables revealed statistically significant positive correlations at the *p* < 0.001 level for all the variables. The correlation coefficients ranged from 0.146 to 0.209, indicating that the measured constructs were meaningfully interrelated and theoretically consistent. Additionally, the descriptive statistics by residential region (Table 3) showed that the mean scores of all the key variables were more than 3.5, indicating a moderate-to-high response level. Furthermore, the standard deviations ranged from 0.44 to 0.84, suggesting stable variance across items, and skewness and kurtosis ranged from –0.874 to 0.060 and –0.068 to 1.111, respectively. These values were within the acceptable thresholds (|skewness| < 1 and |kurtosis| < 1.5), as recommended by West, Finch, and Curran [20], indicating that the variables approximated a normal distribution. When considered together, the significance of the correlation structure, normality of distributions, and stability of variance suggest that the dataset satisfies the SEM’s basic statistical assumptions. Therefore, these data were considered appropriate for SEM-based analyses.

### 3.2. Model Testing

#### 3.2.1. Measurement Model Validation

In this study, a model was specified to examine the path relationships among three latent variables (measured using 10 observed variables) and one observed variable. Prior to the structural model evaluation, CFA assessed whether the 10 measurement variables adequately represented the latent constructs of Health and Fitness Awareness, Health Behavior Action, and Improvement in QOL (Table 4). Results indicated that the measurement model demonstrated an acceptable fit to the data: χ^2^ (degrees of freedom (df) = 39, *p* < 0.001) = 1008.305, CFI = 0.932, TLI = 0.904, RMSEA = 0.061 (90% confidence interval (CI) [0.058, 0.065], *p* < 0.001), and SRMR = 0.046. The standardized factor loadings ranged from 0.233 to 0.944 and were all statistically significant at or below the 0.05 level.

Furthermore, both average variance extracted (AVE) and construct reliability (CR) were assessed to evaluate convergent validity. According to Fornell and Larcker (1981) [21], AVE values above 0.50 indicate adequate convergent validity, and CR values above 0.70 [22] reflect acceptable reliability.

As shown in Table 4, all standardized factor loadings derived from the CFA ranged from 0.233 to 0.944 and were statistically significant at *p* < 0.001. For Health and Fitness Awareness, two items—perceived physical fitness (0.944) and perceived health status (0.813)—showed high factor loadings. In particular, perceived health status showed a non-standardized estimate (B) of 0.837, standard error (SE) of 0.027, and critical ratio (CR) of 30.889, indicating a highly significant effect (*p* < 0.001).

For Health Behavior Action, when regular physical activity was the reference item, all indicators—rest and sleep (0.672), diet and nutrition (0.739), and non-smoking and non-drinking (0.233)—recorded statistically significant factor loadings. The CRs for rest, sleep, diet, and nutrition were 22.726 and 22.317, respectively, confirming their central role as key indicators of this latent construct.

Finally, all items in the Improvement in QOL domain indicated strong loading. When physical health (0.542) was the reference indicator, mental health (0.513), support in daily life (0.635), and reduction in medical expenses (0.603) were significant at *p* < 0.001. Moreover, support in daily life (CR = 29.742) and a reduction in medical expenses (CR = 29.312) recorded particularly high statistical significance.

These results confirm that the measurement model is theoretically sound and valid. In other words, the construct validity of the measurement model was sufficiently supported.

To assess discriminant validity, this study adhered to the criteria proposed by Fornell and Larcker [21], according to which the AVE of each latent variable should exceed the squared correlations (r^2^) between that construct and any other latent construct. As shown in Table 5, the AVE for Health and Fitness Awareness (0.983) was greater than its squared correlations with Health Behavior Action and Improvement in QOL (0.029 and 0.030, respectively). Similarly, the AVE for Health Behavior Action (0.850) exceeded its squared correlations with Health and Fitness Awareness and Improvement in QOL (0.029 and 0.044, respectively). Finally, the AVE for Improvement in QOL (0.906) surpassed the corresponding r^2^ values for the other constructs (0.030 and 0.044). These results indicate that for all latent variables, the AVE values were higher than the squared inter-construct correlations. Therefore, the measurement model used in this study had satisfactory discriminant validity.

#### 3.2.2. Structural Model Validation

In this study, the structural relationships among Health and Fitness Awareness, Health Behavior Action, Safety Behavior Practice, and Improvement in QOL were examined. All the model fit indices indicated an acceptable level of fit: χ^2^ (df = 39, *p* < 0.001) = 3204.676, CFI = 0.929, TLI = 0.867, RMSEA = 0.068 (90% CI [0.066, 0.070], *p* < 0.001), and SRMR = 0.046. As indicated by the path analysis results (Table 6), Health and Fitness Awareness had a significant positive effect on Health Behavior Action (β = 0.468, *p* < 0.001) and Safety Behavior Practice (β = 0.247, *p* < 0.001). In turn, Health Behavior Action significantly influenced Improvement in QOL (β = 0.209, *p* < 0.001); Health and Fitness Awareness had a direct positive effect on QOL (β = 0.196, *p* < 0.001) as well. Safety Behavior Practice had a significant positive effect on Improvement in QOL (β = 0.147, *p* < 0.001), and all path coefficients were statistically significant.

According to these findings, Health and Fitness Awareness influences Improvement in QOL both directly and indirectly through the mediating effects of Health Behavior Action and Safety Behavior Practice.

### 3.3. Regional Pathway Differences

#### 3.3.1. Configural Invariance

A multigroup SEM analysis was conducted to examine whether the structural pathways differed by residential region. As a preliminary step, a configural invariance test was performed to assess whether the same factor structure and pattern of relationships among latent constructs held across the groups. The model fit indices for the configural model were as follows: χ^2^ (df = 259) = 3372.718, *p* < 0.001, CFI = 0.930, TLI = 0.870, RMSEA = 0.039 (90% CI [0.038, 0.040], *p* < 0.001), and SRMR = 0.046. These results indicate adherence to the configural invariance assumption and clarify that the overall factor structure was equivalent for all three regional groups.

We tested a metric invariance model by constraining the factor loadings of the observed variables to be equal across regional groups. This model was compared with the configural (baseline) model, in which configural invariance had already been established, by examining whether the difference in χ^2^ values between the models was statistically significant relative to an increase in the degrees of freedom. Furthermore, the results indicated that the full metric invariance model did not hold (*p* < 0.05). Therefore, partial constraints were relaxed, and a partial metric-invariance model was tested. The difference in χ^2^ values between the configural and partial metric invariance models was 16.881, with a difference of 10 degrees of freedom. Because this difference was not statistically significant at the 0.05 level, partial metric invariance was established.

#### 3.3.2. Structural Invariance

Once metric invariance was confirmed, a structural invariance test was conducted to examine whether the path coefficients among latent variables differed across residential regions (large cities, mid-sized cities, and rural areas). To this end, a fully constrained structural model, which assumes equal path coefficients for all three groups, was compared with the previously confirmed partial metric invariance model.

The comparison revealed a statistically significant difference in fit between the two models (Δχ^2^ = 144.927, Δdf = 62, *p* < 0.001), indicating that the structural paths differed across groups. To identify the specific paths that varied by group, each path was individually constrained and compared with the partial-metric invariance model.

The results revealed that the path from Health and Fitness Awareness to Safety Behavior Practice (Δχ^2^ = 8.968, Δdf = 2, *p* < 0.001) and that from Safety Behavior Practice to Improvement in QOL (Δχ^2^ = 38.840, Δdf = 2, *p* < 0.001) had statistically significant differences across regions. In contrast, no significant differences were found for the following paths:

– Health and Fitness Awareness→ Improvement in QOL (Δχ^2^ = 1.807, *p* > 0.05)
– Health and Fitness Awareness→ Health Behavior Action (Δχ^2^ = 5.491, *p* > 0.05)
– Health Behavior Action → Improvement in QOL (Δχ^2^ = 1.020, *p* > 0.05)

**Figure 2:**
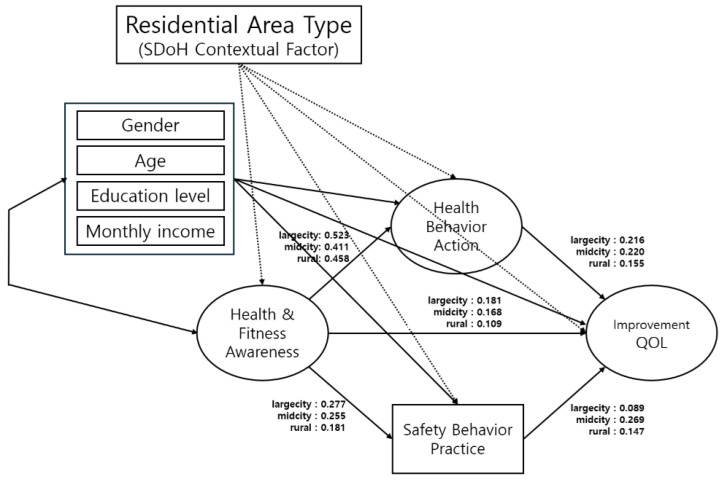
Structural model of regional differences in health behaviour pathways. QOL, quality of life.

According to these findings, the effect of Health and Fitness Awareness on Safety Behavior Practice, as well as that of Safety Behavior Practice on Improvement in QOL, may vary depending on the residential area’s size and its characteristics. Table 7 and Figure 2 depict the path coefficients and regional differences, respectively.

#### 3.3.3. Testing Group Differences in Indirect Effects

Finally, to examine group differences in indirect effects, a bootstrapping analysis was conducted on the metric invariance model without path constraints to assess the significance of the indirect paths within each group. Furthermore, group differences in indirect effects were tested using the Wald test (Table 8). Following the recommendations of Preacher and Hayes [23,24], who suggested using a minimum of 5000 bootstrap samples for accurate estimation and CI calculation, this study used 9000 bootstrap replications at a 95% confidence level to obtain accurate results.

Furthermore, multigroup SEM analysis revealed significant group differences in the two indirect pathways through which Health and Fitness Awareness influenced Improvement in QOL, with one path being mediated by Health Behavior Action and the other by Safety Behavior Practice. In the path of Health and Fitness Awareness→ Health Behavior Action → Improvement in QOL, the indirect effect was highest in the large-sized city group (β = 0.113, 95% CI [0.184, 0.267]), followed by the mid-sized city group (β = 0.090, 95% CI [0.187, 0.292]) and the rural area group (β = 0.071, 95% CI [0.094, 0.225]). Furthermore, the chi-square difference test indicated that the group differences were statistically significant (Δχ^2^ = 5.491, *p* < 0.001).

Similarly, in the Health and Fitness Awareness→ Safety Behavior Practice → Improvement in QOL path, the mid-sized city group exhibited the highest indirect effect (β = 0.069, 95% CI [0.048, 0.092]), followed by the rural area (β = 0.027, 95% CI [0.013, 0.046]) and large-sized city (β = 0.025, 95% CI [0.018, 0.044]) groups. This difference was also statistically significant (Δχ^2^ = 8.968, *p* < 0.001).

These findings suggest that the structure of the indirect pathways through which Health and Fitness Awareness affects Improvement in QOL may vary depending on the regional context. These results emphasize the importance of developing region-specific health promotion and policy implementation strategies.

## 4. Discussion

This study, which is based on SDH theory, adopted a multigroup SEM approach to examine how Health and Fitness Awareness affects Improvement in QOL through the mediating roles of Health Behavior Action and Safety Behavior Practice, and to clarify whether these pathways differ across regions. Results revealed that Health and Fitness Awareness significantly influenced both mediators, which, in turn, had positive effects on Improvement in QOL. Additionally, the indirect pathways from Health and Fitness Awareness to Improvement in QOL were statistically significant. Multigroup analysis further confirmed that the structure of these relationships varied significantly depending on regional size.

This section discusses the theoretical interpretations and implications of the key findings. First, this study empirically confirmed the mediating structure through which Health and Fitness Awareness influences Improvement in QOL. SEM results show that Health and Fitness Awareness positively influences both Health Behavior Action and Safety Behavior Practice. Each of these, in turn, significantly improves QOL. This indicates that awareness of health and fitness affects QOL both directly and indirectly, via engagement in health-related practices.

This result is consistent with earlier research [25,26,27], indicating that individuals’ health perceptions strongly influence their QOL through a complex interplay of structural and individual factors, such as socioeconomic status, health behaviors, and subjective awareness. These findings support policy interventions aimed at strengthening public awareness of health and fitness, as higher awareness is consistently associated with proactive health behaviors and more positive perceptions of QOL.

In this respect, Park and Boo [28] identified factors that are positively associated with subjective health awareness, including regular meals, physical activity, higher educational attainment, adequate sleep, and social engagement, and clarified that injuries sustained during exercise negatively affect health perception. Therefore, to enhance subjective health awareness and improve QOL, it is essential to encourage everyday health management behaviors and minimize negative factors, such as injury during physical activity. This highlights the necessity of developing systematic prevention and management strategies to address these risks.

Second, this study provides empirical evidence for regional health inequality, a key concept in the SDH framework. Multigroup SEM results revealed significant differences across residential area types (large cities, mid-sized cities, and rural areas) in two structural paths: from Health and Fitness Awareness to Safety Behavior Practice and from Safety Behavior Practice to Improvement in QOL. This suggests that even with similar levels of health awareness, the translation of awareness into practice and improvements in QOL depends on the region’s social and environmental contexts.

These results align with the argument by Wilkinson and Marmot [29], emphasizing how health disparities arise from differences in physical environments and resource accessibility. Similarly, Almeida et al. [30] reported that access to living conditions and social resources significantly affects health behaviors and quality-of-life outcomes. Our findings extend Marmot and Allen’s [31] concept of the “causes of the causes” by empirically demonstrating distinct behavioral pathways: Health Behavior Action in large cities and Safety Behavior Practice in mid-sized cities. This shows that structural environments shape health not only as an individual matter but as a socially embedded process.

A study published by Tu et al. [32] analyzing data from 166 countries found that higher access to social and environmental infrastructure was associated with longer healthy life expectancy; however, greater inequality in such access was linked to overall lower health levels. These findings reinforce the interpretation that the regional health inequality patterns identified in this study are closely related to disparities in the distribution of community resources and the structural conditions. Thus, promoting infrastructure equity and regionally tailored policies should be considered essential to achieve health equity.

Third, this study identified clear regional differences in the key mediating factors through which Health and Fitness Awarenessinfluences Improvement in QOL. In large cities, Health Behavior Action was the main mediating factor. In contrast, in mid-sized cities, Safety Behavior Practice played the dominant role. Thus, although levels of health awareness may be similar, the mechanism by which awareness improves QOL differs across regional contexts. This structural variation directly reflects the disparities in Social Determinants of Health (SDoH) resources and the differential exposure to macro-contextual factors. In metropolitan areas (large cities), where SDoH resources such as exercise programs, sports facilities, and comprehensive health information are relatively abundant and highly accessible, the engagement in everyday health management behaviors (Health Behavior Action) is facilitated and thus directly contributes to improvements in QOL. In contrast, in mid-sized and rural areas, where structural disadvantages persist due to limited access to emergency medical services, lower-density safety infrastructure, and fewer community-based support systems, the perception of health risk is significantly heightened. Consequently, concerns regarding safety during physical activity are magnified, making the proactive engagement in Safety Behavior Practice a critical, foundational prerequisite for QOL improvement.

In other words, residents of large cities focus on how frequently and effectively they exercise, whereas those of smaller cities generally prioritize their safe engagement in physical activity. Hence, the prioritization of practice strategies differs by region, and localized approaches should reflect such differences.

These findings are consistent with those of earlier studies. Kim and Kosma [33] and Nickel and Knesebeck [34] reported that older adults in large cities show higher physical activity participation rates and greater awareness of health management than their counterparts living in smaller cities. This finding supports the significance of the Health Behavior Action pathway recognized in the current study’s metropolitan group. Similarly, Rech et al. [35], Frost et al. [36], and Lee and Shepley [37] noted the importance of perceived safety in facilitating physical activity participation. The current study shares a similar context, indicating that safety assurance is considered a prerequisite for engaging in healthy behaviors in mid-sized and rural areas.

The distinctive contribution of this study lies in its empirical demonstration, through SEM, of significant differences in mediating pathways across regions. By identifying the relative influence of mediating factors according to regional type, the study provides more practical applicability than previous research.

Meanwhile, the finding that Safety Behavior Practice played a significant role in improving the QOL in mid-sized cities can be interpreted with respect to the low social capital levels and limited medical service access that characterize these regions. Injuries or accidents may have a disproportionately greater impact on individuals’ daily lives in mid-sized settings than in large-sized ones. This underscores the salience of preventive safety behaviors and highlights how structural disparities—specifically the lack of infrastructure equity, sufficient access to health education, and reliable emergency response systems—fundamentally shape these perceptions and subsequent health outcomes [10,38,39].

Accordingly, there is an urgent need to develop regionally tailored health policies that account for local conditions [40,41]. For example, in large cities, strategies promoting digital health platforms, accessible exercise facilities, and workplace health programs could reinforce everyday health habits. In mid-sized cities, investments in safety infrastructure, community-based safety education, and emergency medical response systems would be effective. In rural areas, digital health initiatives such as telemedicine and mobile health applications could reduce barriers to access.

Ultimately, efforts to enhance QOL by increasing Health and Fitness Awareness must be grounded in practice-oriented strategies that reflect each region’s social context. Such approaches should go beyond isolated health sector interventions and be connected to broader social initiatives to transform local health culture. As emphasized by the WHO [3] and Hall and Jacobson [42], such a multilayered approach aligns with the core principles of the SDH framework, particularly its efforts to develop context-sensitive strategies to promote community health equity. This also suggests the need for intersectoral collaboration between health, education, and urban planning sectors to address the upstream determinants of health.

In summary, this study empirically identified the structural pathways through which Health and Fitness Awareness influences Improvement in QOL under the mediation of Health Behavior Action and Safety Behavior Practice, and clarified that these pathways function differently depending on the residential area’s size. By focusing on region-specific mechanisms, this study deepens theoretical understanding of health inequality and provides concrete guidance for developing tailored health intervention strategies.

This study had several limitations. First, owing to its cross-sectional design, this study could not establish definitive causal relationships among the variables. A longitudinal design would allow researchers to track changes in health behaviors and QOL over time and to examine the temporal ordering of variables, thereby providing stronger evidence for causal pathways. Second, the use of self-reported survey data might have introduced bias stemming from participants’ subjective perceptions, which might have affected response accuracy. Hence, future studies should incorporate objective measures, such as biometric indicators or behavioral tracking data, to enhance methodological rigor. Third, regions were classified based on administrative boundaries; however, these boundaries may not comprehensively reflect the living environments or infrastructure access levels. Hence, future research should use more refined spatial variables or conduct analyses based on functional living zones to better capture the contextual realities of various regions. Fourth, despite the stratified sampling design, potential self-selection bias in survey participation cannot be ruled out, as individuals who agreed to participate may differ systematically from those who did not. Finally, cultural and institutional characteristics specific to South Korea may limit the generalizability of these findings to other countries. Future studies should examine whether similar structural pathways are observed in different cultural contexts.

## 5. Conclusions

Grounded in the SDH framework, this study demonstrated that Health and Fitness Awareness affects QOL both directly and indirectly through Health Behavior Action and Safety Behavior Practice, with the mediating role differing by regional size. Specifically, Health Behavior Action was more influential in large cities, whereas Safety Behavior Practice was more prominent in mid-sized cities.

These findings highlight the need for regionally tailored strategies to reduce health inequalities. Strengthening safety practices in mid-sized cities, expanding structural support in large cities, and enhancing health literacy and community education across all regions are critical for advancing health equity.

## Figures and Tables

**Table 1:** Demographic characteristics of participants.

Variable	Category	Frequency	Percentage
Gender	Male	3417	51.8
	Female	3184	48.2
Age group (year)	10s–20s	1592	24.1
	30s–40s	1998	30.3
	50s–60s	2162	32.8
	70 and above	849	12.9
Educational level	High school or below	1306	19.8
	Bachelor’s degree	2293	34.7
	Graduate degree or higher	3002	45.5
Monthly income ($)	Less than 1540	515	7.8
	Approximately 1540–3080	1944	29.5
	Approximately 3080–4620	2630	39.8
	More than 4620	1512	22.9
Region	Large city	2937	44.5
	Mid-sized city	2134	32.3
	Rural area	1530	23.2
Total		6601	100.0

**Table 2:** Descriptive statistics of the variables.

Variable	Health and Fitness Awareness	Health Behavior Action	Safety Behavior Practice	Improvement in QOL
Health and Fitness Awareness	1	–	–	–
Health Behavior Action	0.171 (*p* < 0.001)	1	–	–
Safety Behavior Practice	0.198 (*p* < 0.001)	0.146 (*p* < 0.001)	1	–
Improvement in QOL	0.172 (*p* < 0.001)	0.209 (*p* < 0.001)	0.196 (*p* < 0.001)	1

QOL, quality of life.

**Table 3:** Correlations among the variables.

City Size	Variable	Mean	SD	Skewness	Kurtosis
Large-sized city (*n* = 2937)	Health and Fitness Awareness	3.73	0.688	–0.65	0.81
	Health Behavior Action	3.59	0.552	–0.42	0.22
	Safety Behavior Practice	3.73	0.836	–0.87	1.11
	Improvement in QOL	3.89	0.448	–0.21	–0.07
Mid-sized city (*n* = 2134)	Health and Fitness Awareness	3.7	0.763	–0.47	0.19
	Health Behavior Action	3.53	0.602	–0.33	0.1
	Safety Behavior Practice	3.69	0.771	–0.56	0.45
	Improvement in QOL	3.88	0.479	–0.12	0.13
Rural area (*n* = 1530)	Health and Fitness Awareness	3.69	0.753	–0.55	0.59
	Health Behavior Action	3.52	0.574	–0.29	–0.01
	Safety Behavior Practice	3.68	0.819	–0.63	0.59
	Improvement in QOL	3.89	0.49	0.06	–0.01

SD, standard deviation; QOL, quality of life.

**Table 4:** Results of confirmatory factor analysis.

Latent Variable		Indicator	Standardized Estimate	Estimate	SE	CR	*p*-Value
Health and Fitness Awareness	→	physical_status_rec	0.944	1	–	–	–
	→	health_status_rec	0.813	0.837	0.027	30.889	<0.001 ***
Health Behavior Action	→	regular_phy_act	0.361	1	–	–	–
	→	rest_sleep	0.672	1.633	0.072	22.726	<0.001 ***
	→	nutritional_sup	0.739	1.725	0.077	22.317	<0.001 ***
	→	drink_smoke	0.233	0.83	0.06	13.723	<0.001 ***
Improvement in QOL	→	physical_health	0.542	1	–	–	–
	→	mental_health	0.513	1.087	0.04	27.151	<0.001 ***
	→	life_help	0.635	1.425	0.048	29.742	<0.001 ***
	→	healthcare_cost	0.603	1.439	0.049	29.312	<0.001 ***

Note: *** *p* < 0.001; CR, critical ratio; SE, standard error; QOL, quality of life.

**Table 5:** Assessment of discriminant validity.

Variable	1. Health and Fitness Awareness	2. Health Behavior Action	3. Improvement in QOL	AVE
1. Health and Fitness Awareness	1	–	–	0.983
2. Health Behavior Action	0.171 (*p* < 0.001)	1	–	0.85
3. Improvement in QOL	0.172 (*p* < 0.001)	0.209 (*p* < 0.001)	1	0.906

AVE, average variance extracted.

**Table 6:** Results of path analysis.

Pathway	Standardized Coefficient	Estimate	SE	CR	*p*-Value
Health and Fitness Awareness → Health Behavior Action	0.468	0.22	0.013	17.124	<0.001 ***
Health and Fitness Awareness → Safety Behavior Practice	0.247	0.296	0.02	14.468	<0.001 ***
Health Behavior Action → Improvement in QOL	0.209	0.183	0.019	9.408	<0.001 ***
Health and Fitness Awareness→ Improvement in QOL	0.196	0.081	0.01	8.023	<0.001 ***
Safety Behavior Practice → Improvement in QOL	0.147	0.05	0.005	9.353	<0.001 ***

Note: *** *p* < 0.001; CR, critical ratio; SE, standard error; QOL, quality of life.

**Table 7:** Test of homogeneity between groups.

Model	χ^2^	df	Δχ^2^	Δdf	*p*-Value
Configural Invariance Model	3372.718	306	–	–	<0.001 ***
Partial Metric Invariance Model	3389.599	316	16.881	10	<0.001 ***
Structural Invariance Model	3534.526	378	144.927	62	<0.001 ***
Health and Fitness Awareness→ QOL	3391.406	318	1.807	2	<0.001 ***
Health and Fitness Awareness→ Health Behavior Action	3395.09	318	5.491	2	<0.001 ***
Health and Fitness Awareness→ Safety Behavior Practice	3398.567	318	8.968	2	<0.001 ***
Health Behavior Action → QOL	3390.619	318	1.02	2	<0.001 ***
Safety Behavior Practice → QOL	3428.439	318	38.84	2	<0.001 ***

Note: *** *p* < 0.001; df, degree of freedom; QOL, quality of life.

**Table 8:** Indirect and group-specific indirect effects.

Pathway	Indirect Effect (β) 95%	Confidence Interval (CI)	Between-Group Difference (B)
Health and Fitness Awareness→ Health Behavior Action → QOL	Large city: 0.113	[0.184, 0.267]	5.491 ***
	Mid-sized city: 0.090	[0.187, 0.292]	
	Rural area: 0.071	[0.094, 0.225]	
Health and Fitness Awareness→ Safety Behavior Practice → QOL	Large city: 0.025	[0.018, 0.044]	8.968 ***
	Mid-sized city: 0.069	[0.048, 0.092]	
	Rural area: 0.027	[0.013, 0.046]	

Note: *** *p* < 0.001

## Data Availability

Publicly available datasets were analyzed in this study. These data can be found at the Korea Disease Control and Prevention Agency (KDCA) website: https://www.kdca.go.kr/ (accessed on 15 May 2025).

## Abbreviations

The following abbreviations are used in this manuscript:

QOL	Quality of Life
SDH	Social Determinants of Health
SEM	Structural Equation Modeling
